# Gut Microbiome and Intestinal Colonization with Multidrug-Resistant Strains of Enterobacterales: An Interplay Between Microbial Communities

**DOI:** 10.3390/antibiotics14090890

**Published:** 2025-09-03

**Authors:** Béla Kocsis, Dóra Szabó, László Sipos

**Affiliations:** 1 Institute of Medical Microbiology, Semmelweis University, 1089 Budapest, Hungary; szabo.dora@semmelweis.hu; 2 Department of Neurosurgery and Neurointervention, Semmelweis University, 1085 Budapest, Hungary; 3 HUN-REN-SU Human Microbiota Research Group, 1052 Budapest, Hungary

**Keywords:** gut microbiome, intestinal colonization, multidrug-resistant Enterobacterales, carbapenem-resistant *Klebsiella pneumoniae*, carbapenem-resistant *Escherichia coli*

## Abstract

**Background**: The intestinal tract is a host to a high number of diverse bacteria, and the presence of multidrug-resistant (MDR) Enterobacterales strains acts as a reservoir and a source of infection. The interactions between the intestinal microbiome and colonizer Enterobacterales strains influence long-lasting colonization. **Aims**: In this narrative review, we summarize available data about the intestinal colonization of MDR Enterobacterales strains and correlations between colonization and the intestinal microbiome. **Results**: Several endogenous and exogenous factors influence the intestinal colonization of MDR Enterobacterales strains. On the gut microbiome level, the intestinal microbial community is composed of the Lachnospiraceae family (e.g., *Lachnoclostridium*, *Agathobacter*, *Roseburia*, *Tyzzerella*), which indicates a protective role against colonizer MDR Enterobacterales strains; by contrast, a high abundance of Enterobacterales correlates with the colonization of MDR Enterobacterales strains. In specific patient groups, striking differences in microbiome composition can be detected. Among hematopoietic stem-cell-transplanted patients colonized by extended-spectrum beta-lactamase (ESBL)-producing Enterobacterales, a greater abundance of *Bifidobacterium*, *Blautia*, *Clostridium*, *Coprococcus*, *L-Ruminococcus*, *Mogibacteriaceae*, *Peptostreptococceae* and *Oscillospira* was observed compared to patients not colonized by ESBL-producing strains, who had a greater abundance of Actinomycetales. In liver transplant patients, a reduction in the alpha-diversity of the intestinal microbiome in fecal samples correlates with the carriage of MDR Enterobacterales. **Conclusions**: Intestinal colonization with MDR Enterobacterales is a multifactorial process that involves the MDR strain (e.g., its plasmids, fimbria), host and mucosal factors (e.g., IgA and defensin) and exogenous factors (e.g., use of antibiotics, hospitalization). On the gut microbiome level, the Lachnospiraceae family is dominant among intestines not colonized by MDR strains, but a high abundance of Enterobacterales was correlated with colonization with MDR Enterobacterales strains.

## 1. Introduction

Intestinal colonization with multidrug-resistant (MDR) Enterobacterales strains represents a source of infection, and the transmission from person to person takes place through contaminated carriers [1,2]. Among MDR Enterobacterales strains, *Klebsiella pneumoniae* and *Escherichia coli* are the most frequently reported pathogens, and they colonize the intestinal tract asymptomatically. However, these MDR pathogens are responsible for a high number of difficult-to-treat infections, namely urinary tract infections, bloodstream infections and wound infections [3]. These infections are great challenges worldwide because a limited number of effective antibiotics are available to treat such infections; therefore, the treatment for these infections is complicated and entails a longer hospital stay and a greater burden on hospitals. Furthermore, infections caused by MDR Enterobacterales strains lead to higher mortality rates [4]. It has been estimated that approx. 4.7 million patients die each year from MDR bacterial infections worldwide, and this number increases annually [5]. The most important medically relevant MDR bacteria are listed in the World Health Organization priority list, and the critical group on this list comprises both carbapenem-resistant and third-generation cephalosporin-resistant Enterobacterales [6].

MDR Enterobacterales strains circulate between different niches, including human hosts, animals and the environment [7,8]. In all these niches, Enterobacterales strains can proliferate, persist and survive, and these strains can be passed on from one host to another. Furthermore, these features enhance the development of multidrug resistance, because bacterial strains can take up and accumulate different antibiotic resistance genes from the surrounding bacteria in different hosts, and thus multidrug-resistant strains can evolve and colonize the intestinal tract for a longer period of time. The intestinal tract serves as an excellent space for bacteria to develop and to maintain antibiotic resistance [2].

The gastrointestinal tract is a huge mucosal surface of the human body and is inhabited by a high density of different microbes. The major role of these high-density microbial communities in the intestinal tract is to protect the human host from bacterial pathogens that can colonize the mucus layer, and later on these colonizer bacteria may invade the tissues. This protective effect is also called colonization resistance [9,10]. However, the human intestinal tract is an enormous reservoir of diverse bacterial species and can host antibiotic-resistant bacteria in high numbers. Apart from asymptomatic colonization on the intestinal mucus layer, MDR bacteria can act as transmitters of resistance genes. Through horizontal gene transfer, extended-spectrum beta-lactamase (ESBL) genes (e.g., *bla*_SHV_, *bla*_CTX-M_), carbapenemase genes (e.g., *bla*_KPC_, *bla*_NDM_, *bla*_VIM_ and *bla*_OXA-48_) and other associated resistance genes can be transferred between different bacterial species in the intestine [11].

Several factors influence the intestinal colonization and transfer of MDR strains in the human gut, including hospitalization, prior antibiotic therapy, dysbiosis and underlying diseases. Furthermore, travel to geographical regions that face a high prevalence of MDR bacteria can also be considered a risk factor for acquiring and carrying MDR bacteria in the human intestinal tract.

The aim of this narrative review is to summarize data about the intestinal colonization of MDR Enterobacterales strains, to give an update on the epidemiology of MDR Enterobacterales colonization and to analyze the role of the intestinal microbiome and additional factors in colonization. The literature selection was created based on published scientific articles from Pubmed, Scopus and Web of Science. The inclusion criteria were clinical studies and murine models reporting on intestinal colonization with MDR Enterobacterales. Non-peer-reviewed articles and articles not published in English were excluded.

## 2. Global and Setting-Specific Epidemiology of Intestinal Colonization with MDR Enterobacterales

Different countries have report on a diverse rate of intestinal colonization of MDR Enterobacterales. Interestingly, a carriage rate difference is also seen between different patient groups and the general population. It should be noted that the screening of intestinal carriage is commonly performed among hospitalized patients, but less frequently in the general population. During the last two decades, an eight-fold increased rate of intestinally carried ESBL-producing *E. coli* has been reported generally worldwide. Interestingly, an increase has been seen in the community, in the general population and in the healthcare setting among hospitalized patients [12,13]. Among colonizer MDR Enterobacterales strains, the ESBL-producing *K. pneumoniae* and *E. coli* are highly prevalent; however, in several countries, the carbapenemase-producing *K. pneumoniae* and *E. coli* have also frequently been detected [14].

During a study in Thailand at an intensive care unit (ICU), 134 patients out of 215 were colonized by ESBL-producing Enterobacterales. The dominant MDR strains were ESBL-positive *E. coli* (67.5%) and *K. pneumoniae* (19.4%) According to molecular typing, ST131, ST648, ST38, ST393 and ST1193 were prevalent among colonizing MDR *E. coli* clones [15].

Based on routine screening practice in Switzerland, an 8% intestinal colonization with ESBL-producing Enterobacterales was reported among patients at admission to ICU. Among colonizing strains, the ESBL-producing *E. coli* dominated, followed by *K. pneumoniae* and *Providentia stuartii*. Interestingly, among 24 intestinally colonized patients, 3 developed an infection caused by an ESBL-producing strain [16].

During active surveillance at an ICU in France, a 5.3% prevalence of intestinal colonization with EBSL-producing Enterobacterales strains was reported. Altogether, twenty-eight patients were colonized by 29 strains. ESBL-producing *E. coli* was dominant, followed by *K. pneumoniae*, *Enterobacter* spp. and *Citrobacter sedlakii* [17].

A study in Spain reported 16% (41/254) of carbapenemase-producing Enterobacterales colonization in ICU patients, and OXA-48-producing *K. pneumoniae* was the most commonly isolated. Additionally, 17.7% (45/254) of patients were colonized by ESBL-producing Enterobacterales [18]. In a study among pre-term newborns in Serbia between 2017 and 2018, a remarkable high prevalence (59%) of intestinally carried ESBL-producing *K. pneumoniae* and ESBL-producing *E. coli* was detected [19].

A report about newborns hospitalized in a neonatal ICU in Morocco between 2013 and 2015 described 59.4% intestinal colonization with ESBL-producing Enterobacterales; however, a 12.5% colonization rate of carbapenemase-producing Enterobacterales was detected [20]. In China, a study in 2019 reported a colonization rate of about 8.6% for carbapenemase-producing Enterobacterales among hospitalized pediatric patients [21]. Several studies from different countries from 2010 to 2018 reported on a diverse intestinal colonization rate of ESBL-producing Enterobacterales in pediatric patients hospitalized in different clinical departments, as follows: Tunisia, 28%; Gabon, 45%; Cambodia, 55%; and Tanzania, 56% [22,23,24,25]. Interestingly, among healthy individuals, the intestinal carriage rate of Enterobacterales strains resistant to extended-spectrum cephalosporins in Tunisia was shown to be increasing with time. Between 2009 and 2010, a 7.3% rate was detected; however, between 2021 and 2023, this was 9.4% among healthy volunteers. Among the isolated MDR strains, *K. pneumoniae* and *E. coli* were the most prevalent, and CTX-M-15 type beta-lactamase was the most common resistance determinant [26]. A high rate of carbapenem-resistant Enterobacterales was reported from rectal swabs in Israel. Both KPC- and NDM-producing Enterobacterales strains were detected [27]. Global epidemiological data about intestinal colonization with MDR Enterobacterales are summarized in Table 1.

## 3. Host and Microbiome Mechanisms in Intestinal Colonization with MDR Enterobacterales

**Figure 1:**
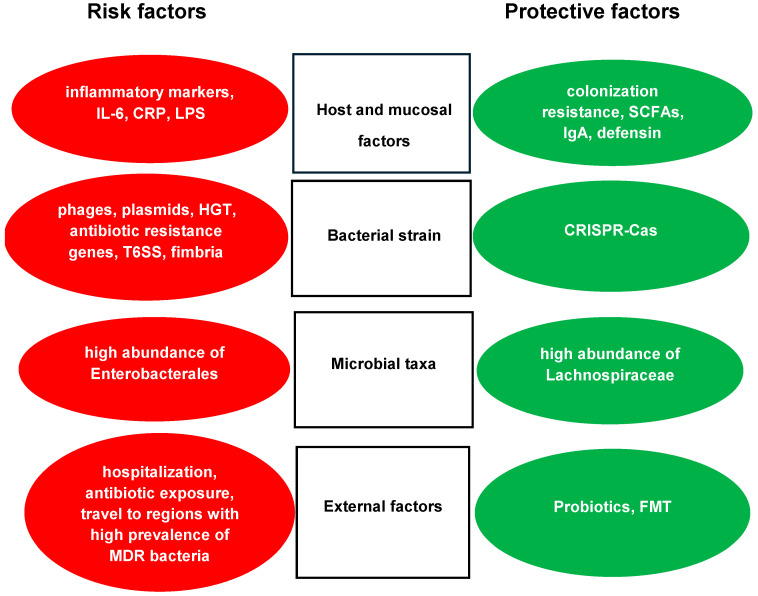
Factors influencing intestinal colonization with MDR Enterobacterales strains. Circles in red indicate risk factors that enhance dysbiosis and intestinal colonization with MDR Enterobacterales; circles in green show protective factors that diminish colonization with MDR Enterobacterales. Abbreviations: IL-6: Interleukin-6. CRP: C-reactive protein. LPS: Lipopolysaccharide. SCFAs: Short-chain fatty acids. HGT: Horizontal gene transfer. HGT is an effective mechanism among bacterial cells to take up genetic materials (e.g., plasmids, phages) from other bacteria. T6SS: Type VI secretion system. T6SS is an effector mechanism that releases proteins from the bacterial cell. It enhances colonization and increases competition between bacteria. CRISPR-Cas: Clustered regularly inter-spaced short palindromic repeats. CRISPR-Cas is a protective mechanism for the bacterium against external nucleic acids. FMT: Fecal microbiota transplantation.

Intestinal colonization with MDR Enterobacterales is a multifactorial process. The intestinal microbiome, host factors and immune status of the patient, as well as the colonizer strain itself and its resistance plasmids, influence a successful short- or long-term intestinal colonization (Figure 1).

The intestinal tract normally possesses colonization resistance that protects the human body from pathogenic bacteria, but MDR Enterobacterales strains (e.g., *K. pneumoniae*, *E. coli*) can easily colonize the mucus layer because these bacterial species are normal inhabitants of the human intestine. However, through horizontal gene transfer (e.g., acquisition of plasmids), the antibiotic-resistant genes from MDR strains can be transmitted and exchanged between different bacterial species in the intestinal tract. Therefore, a higher abundance of Enterobacterales enhances longer-term MDR colonization. The increase in the abundance of MDR Enterobacterales strains can be influenced by mucosal factors (e.g., IgA, defensin production); furthermore, the different plasmids harboured by the MDR strains (e.g., IncF, IncL) influence IgA and defensin levels in the intestine [39] and, additionally, small cryptic plasmids in MDR *E. coli* and *K. pneumoniae* can also play a role in intestinal colonization with MDR strains [40]. The expression of type 3 fimbria through sensor histidine kinase CpxA in *K. pneumoniae* influences colonization in the intestinal mucus layer [41]. Furthermore, Type VI Secretion System (T6SS) also helps the intestinal colonization of *K. pneumoniae*, as well as the translocation of bacterium from the intestinal tract to the bloodstream [42].

Hospitalization, invasive interventions, the application of antibiotics, induced collateral damage in the intestinal microbiota and dysbiosis all enhance the intestinal colonization of the MDR Enterobacterales strain. Furthermore, antibiotic exposure has multiple effects on colonization, because the bacterial cell count, bacterial diversity and the antibiotic resistance gene copy number can be influenced [38]. Travel to countries with a high prevalence of MDR bacteria also indicates a risk factor for acquiring MDR Enterobacterales strains, which can mainly be explained by exogenous factors (e.g., dietary factors, uncooked vegetables, contaminated water) [43].

On the microbiome level, several bacterial taxa have been detected that have a protective role against MDR colonization, namely *Lachnospiraceae*, *Dorea*, *Atopobiaceae*, *F. prausnitzii*, *Collinsella aerofaciens*, *Roseburia* and *Tyzzerella.* These taxa were abundant in individuals never colonized by MDR Enterobacterales strains [30]. However, upon acquisition of MDR Enterobacterales strains, a higher abundance of Enterobacterales was detected in the intestinal microbiome [43]. It can be assumed that the colonization of MDR Enterobacterales strain is a correlation of different microbial communities in the intestinal tract that is influenced by different endogenous and exogenous factors.

### Additional Factors in Intestinal Microbial Communities

Several biomarkers were detected that play a role in chronic inflammation in the intestinal mucosa during dysbiosis. The most important biomarkers are interleukin (IL-6), c-reactive protein (CRP) and lipopolysaccharide (LPS). IL-6 and CRP are well-known proinflammatory markers; however, IL-10 has an anti-inflammatory function. In the case of LPS, this is a major component in the Gram-negative cell wall, as it has a proinflammatory role and can induce systemic inflammation in the human body [44].

Short-chain fatty acids (SCFAs) (e.g., acetate, propionate and butyrate) also play a role in mucosal immunity through having a G-protein-receptor-related function or acting through histone deacetylase activity. SCFAs are commonly produced by commensal bacteria in the intestinal tract, such as *Faecalibacterium* spp. and *Bifidobacterium* spp. [45].

It has already been established that probiotic bacteria have beneficial roles in the intestinal microbiota. The most important and most frequently analyzed probiotic bacteria are *Lactobacillus* spp., *Bifidobacterium* spp. and *Streptococcus* spp. These bacteria have important roles, such as antagonism against pathogenic bacteria, synthesis of beneficial nutrients, protection of mucosal integrity, antiallergenic features and the synthesis of antimicrobial substances and bacteriocins. Furthermore, the production of SCFAs is also a major role of probiotic bacteria [44]. Apart from probiotic bacteria, it has also been demonstrated that some fungi can be normally present in the intestinal microbiota, namely *Candida* spp., Cladosporium, *Aureobasidium*, *Aspergillus* spp. and *Saccharomyces cerevisiae* [44].

Bacteriophages (or phages) are viruses that can infect bacteria and replicate inside the bacterial cell. It has been demonstrated in several studies that bacteriophages play a central role in horizontal genetransfer between different bacterial cells. This can take place in the intestinal tract with high efficacy because of the gut host’s high density of diffrerent bacterial species. However, bacterial cells have different mechanisms that control or regulate the uptake of genetic materials. The most important mechanism is the CRISPR-Cas system, which is composed of several chromosomal gene sequences and proteins [46].

Additionally, a study demonstrated that different commensal *E. coli* strains can inhibit the growth of MDR *E. coli* strain based on different carbohydrate consumption and metabolic activity in the intestinal tract [47].

## 4. Hospitalization as a Factor in Intestinal Colonization with MDR Enterobacterales

Several earlier studies reported on a higher prevalence of intestinal MDR Enterobacterales carriage among hospitalized patients compared to that for people in the community [12,48,49,50]. This has been explained by factors in correlation with hospitalization, such as the use of different broad-spectrum antibiotics, induced collateral damage in the intestinal normal flora during treatment, dysbiosis and direct person-to-person transmission (e.g., from patient to patient), as well as hospital environmental factors (e.g., water, food) that can enhance intestinal colonization with MDR Enterobacterales strains [51,52].

Intestinal colonization with MDR Enterobacterales involves both the gut microbiota and the colonizer MDR strain. All factors that can disrupt the endogenous intestinal microbiota can enhance the effective colonization of the exogenous MDR strain [53]. This colonization starts asymptomatically, and may last for a longer period of time, but it can be both a reservoir and a source of MDR strains [54]. It has been demonstrated previously in a follow-up study among newborns in Sweden that initial intestinal colonization by ESBL-producing *K. pneumoniae* can lead to simultaneous co-colonization with an ESBL-producing *E. coli* strain, thus indicating a role of ESBL-producing *K. pneumoniae* as a source of resistance genes in the intestinal tract. Therefore, through horizontal gene transfer, the transmission of resistance genes can take place [32].

Furthermore, prior asymptomatic intestinal colonization by MDR *K. pneumoniae* increases the incidence of systemic infections (e.g., bloodstream infections) caused by MDR *K. pneumoniae* strains, indicating the successful translocation of the earlier colonizing strain from the intestine to the bloodstream [55,56,57].

This transmission and translocation of MDR Enterobacterales can lead to very severe conditions in patient groups with high susceptibility to infections (e.g., immunocompromised patients), such as newborns, hematopoietic stem cell transplant patients and patients in long-term care facilities. Among newborns, several risk factors for colonization have been reported, namely level of immaturity, hospitalization in neonatal intensive care unit, invasive procedures [35], low birth weight, low gestational age, mother-to-newborn transmission and prior treatment with antibiotics [32,58,59].

On the other hand, postinfection colonization can also occur [60]. This has been demonstrated in a study where two patients were intestinally colonized with the same strain of ESBL-producing *E. coli* that had earlier caused a urinary tract infection (UTI) in that patient. This was proven by a one-year follow-up test of fecal samples from the patients. Interestingly, in these two patients, exclusively the same ESBL-producing *E. coli* strain was detected in the follow-up test; by contrast, in a third patient, different ESBL-producing *E. coli* and *K. pneumoniae* strains were isolated in the fecal samples [40]. These results indicate that a longer-term (12 months) asymptomatic intestinal colonization after a recovery from a UTI is possible with the same ESBL-producing *E. coli* strain that caused the earlier infection; however, in the intestinal tract, diverse MDR strains can also evolve through horizontal gene transfer.

### 4.1. Intestinal Colonization Among Patients in the Intensive Care Unit

Hospitalization in an intensive care unit (ICU) has also been associated with the acquisition of intestinally carried ESBL-producing Enterobacterales strains. It has been reported that between 5 and 10% of patients were colonized with ESBL-producing Enterobacterales after treatment in an ICU; this correlated with a higher frequency of infections caused by ESBL-producing Enterobacterales, and these infections were associated with higher mortality rates [61].

Intestinal colonization among patients in an ICU was determined in Lao PDR. Altogether, 137 patients were included in this study, and ESBL-producing *E. coli*, *K. pneumoniae* and carbapenem-resistant Enterobacterales strains were targeted. An overall 17.5% colonization rate for ESBL-producing *E. coli* and 3.6% carbapenem-resistant Enterobacterales was determined. NDM-1- and CTX-M-15-producing *K. pneumoniae* were dominant among carbapenem-resistant strains; moreover, a high-risk clone of *K. pneumoniae* ST147 was also detected among colonizer strains [38].

### 4.2. Intestinal Colonization Among Patients in a Long-Term Care Facility

In a recent study in a long-term care facility (LTCF) examining 187 hospitalized patients during a one-year period in Spain, it was demonstrated that ESBL-producing *E. coli* was the dominant colonizer strain among MDR bacteria. Interestingly, 51.4% of the individuals at LTCF were intestinally colonized with ESBL-producing *E. coli*; by contrast, 48.6% of patients hospitalized at LTCF were never colonized by ESBL-producing *E. coli*. However, among colonized patients, 15.5% were persistently colonized and 35.8% of individuals were intermittently colonized. An antibiotic exposition on 187 hospitalized individuals was documented and the most frequently prescribed antimicrobial agents were amoxicillin plus clavulanic acid, third-generation cephalosporin and fluoroquinolones [33].

### 4.3. Intestinal Colonization Among Hematopoietic Stem Cell Translant Recipients

High rates of intestinal colonization with carbapenem-resistant *K. pneumoniae* and *E. coli* were reported among hematopoietic stem cell transplant recipients in Tunisia during a study between 2015 and 2019. NDM-producing *K. pneumoniae* and OXA-48 producing *E. coli* were the predominant MDR Enterobacterales strains. Altogether, eighty-one episodes of carbapenem-resistant Enterobacterales colonization were detected in fifty-five patients, who were diagnosed mainly with acute leukemia and aplastic anemia [37].

### 4.4. Microbiome Diversity in Leukemia Patients Colonized by MDR Enterobacterales

Among leukemia patients, the leading cause of death is bacteremia [62] and it has previously been established that the major source of causative agents is the intestinal tract, and usually only a single bacterium invades into the bloodstream from the intestinal microbiota. Therefore, the intestinal microbiome and microbial communities correlate, or the causative agent bacterium should reach a threshold in the intestine to make it possible to enter the circulation. It has been demonstrated in a study about acute myeloid leukemia patients with bacteremia that 7 out of 63 patients had >30% relative abundance, a value considered to be the threshold of domination in the intestinal tract or in the oral cavity, of the causative agent in the intestine that caused the bacteremia. This relative abundance was determined by 16S rRNA sequencing from stool and oral samples [63]. However, in another study, this threshold value was detected only in less than 50% of adult patients diagnosed with bloodstream infection after allogeneic hematopoietic cell transplantation [64]. This relatively low number of confirmed cases of bacteremia caused by bacterial pathogens from the intestinal tract that reached the threshold value can be explained by the fact that other sites in the human body can act as gateways for the bacteria to reach the bloodstream (e.g., lungs, indwelling catheter, etc.) or because of the interplay of microbial communities in the microbiota that interfere with the abundance of bacterial taxa, and as a result affect the penetration and translocation of bacterial pathogens.

### 4.5. Microbiome Diversity in Liver-Transplant Patients Colonized by MDR Enterobacterales

During a study, the intestinal microbiome and colonization of MDR Enterobacterales were analyzed in 195 patients after liver transplantation. The reasons for liver transplantation were hepatitis C virus infection, non-alcoholic fatty liver diseases, alcohol-related liver diseases and hepatocellular carcinoma [65]. Among the patients studied, a remarkably high rate of intestinal colonization with MDR bacteria was reported: 65% of patients were colonized at least once during the one-year study period. The most frequently reported MDR bacteria were third-generation cephalosporin-resistant Enterobacterales (47%) and carbapenem-resistant Enterobacterales (18%). This investigation analyzed the alpha-diversity of the microbiome in fecal samples through 16s rRNA sequencing; among patients colonized by third-generation cephalosporin-resistant Enterobacterales and carbapenem resistant Enterobacterales, interestingly, in both patient groups, a reduction in alpha-diversity was found. Additionally, beta-diversity was also analyzed, and a significant difference was detected between intestinal microbiome colonization by carbapenem-resistant and third-generation cephalosporin-resistant Enterobacterales compared to non-colonized intestinal microbiome samples [65]. It is worth mentioning that patients in this study were exposed to high levels of different antibiotics; moreover, 96% of patients were exposed to multiple antibiotics during this study. Generally, 14-day-long antibiotic exposure enhanced a decrease in gut microbial diversity. Interestingly, third-generation cephalosporins, piperacillin-tazobactam, carbapenems and fluoroquinolones induced lower alpha-diversity; as a contrast, first- and second-generation cephalosporins did not significantly influence the alpha diversity of the intestinal microbiome [65].

The demographic data (e.g., age, ethnicity) of patients did not significantly influence the intestinal microbiome alpha diversity; however, the different underlying liver diseases had a significant impact on alpha diversity. Patients with alcohol-related liver diseases had a significantly lower alpha diversity in the intestinal microbiome compared to patients with other liver diseases. However, patients with hepatocellular carcinoma had a significantly higher alpha diversity compared to patients without cancer. Additionally, patients with alcohol-related liver diseases had a significantly lower bacterial amount of *Faecalibacterium prausnitzii* and had an increased number of *Streptococcus* spp. and *Lactobacillus* spp. [65].

### 4.6. Microbiome Diversity in Hematopoietic Stem Cell Recipients Colonized by MDR Enterobacterales

It has been demonstrated that during allogeneic hematopoietic stem cell transplantation, a significant decrease in intestinal microbiome diversity is seen. This can be explained by many factors such as chemotherapy, prophylactic and therapeutic applications of antibiotic agents or even inflammation in the intestinal mucus layer. However, a specific microbiome difference was detected at the baseline before hospitalization between patients colonized and not colonized by ESBL-producing Enterobacterales strains. Among patients colonized by ESBL-producing strains, a higher abundance of *Bifidobacterium*, *Blautia*, *Clostridium*, *Coprococcus*, *L-Ruminococcus Mogibacteriaceae*, *Peptostreptococceae* and *Oscillospira* was detected; however, among patients not colonized by ESBL-producing strains, a greater abundance of *Actinomycetales*, *Staphylococcus* and *Sutterella* was detected. Interestingly, during the study period, certain patients retained ESBL colonization, but other patients had a negative fecal sample for the ESBL strain. *Akkermansia*, *Dialister*, *Erysipelotrichaceae* and *Methanobrevibacter* had a higher abundance in patients with retained fecal ESBL positivity, compared to patients with a negative ESBL fecal test [34].

### 4.7. Microbiome Diversity in Patients at a Dutch Nursing Home and in the General Population of The Netherlands

In a study about hospitalized patients in a Dutch nursing home, twenty-seven participants were included in the study and 93 fecal samples were analyzed. Interestingly, among detected colonized strains, 27 yielded MDR positivity, and among ESBL-producing bacteria (*n* = 14), the dominant strain was *E. coli* (*n* = 10), followed by *Enterobacter cloacae* (*n* = 3) and *Citrobacter non-koseri* (*n* = 1). Additionally, fluoroquinolone and aminoglycoside-resistant *E. coli* strains (*n* = 13) were also detected. On the microbiome level, 16S rRNA gene sequencing detected no differences in alpha diversity or beta diversity between fecal samples of MDR strain colonized and non-colonized samples. However, an abundance of three bacterial taxa, namely *Dorea*, *Atopobiaceae* and *Lachnospiraceae*, was detected in patients never colonized with an MDR strain during this study [30].

In contrast, during a Dutch cross-sectional population study, asymptomatic intestinal colonization with ESBL-producing bacteria was analyzed, and a 7% (198/2751) prevalence was determined. Among the ESBL-positive strains, 44 were proved to be CTX-M type beta-lactamase-producing *E. coli*. However, based on metagenomic shotgun sequencing for intestinal microbiome analysis, no significant differences were found in the composition of the microbiome between ESBL-positive and ESBL-negative stool samples [31]. This finding suggests that the intestinal microbiome differences are not that huge between ESBL-carriage and ESBL-negative samples in the general population.

## 5. Acquisition of MDR Enterobacterales Strains and Microbiome Change During Travel

The prevalence of MDR Enterobacterales intestinal carriage rate is increasing worldwide. Interestingly, a recent study in Sweden reported on a 5% asymptomatic intestinally carried ESBL-producing Enterobacterales in the Swedish general population. However, this study determined that the main risk factor for acquiring and carrying intestinal MDR bacteria is travel to different geographical regions that have high rates of MDR bacteria (e.g., Asia, Africa) [40,66,67].

The acquisition of ESBL-producing Enterobacterales was reported among Dutch tourists. An overall 34.3% of travellers were found to be positive for intestinally carried ESBL-producing Enterobacterales strains after travel abroad. Interestingly, there were differences in the rate of acquisition of MDR bacteria; after a visit to Southern Asia, 75.1% of travellers were colonized, while travel to different parts of Africa resulted in a maximum 42% colonization rate [68].

Other reports about Swiss and Danish tourists in South Asia (e.g., India, Sri Lanka) have described striking differences in intestinal carriage rates of ESBL-producing *E. coli* before and after travel, namely that 70% of Swiss and >90% of Danish travellers acquired ESBL-producing *E. coli* in the intestine during travel to South Asia [69,70].

It has been demonstrated in a study that during travel, different alterations in the gut microbiome can be detected. Altogether, 368 US international travellers were included in a study and the intestinal microbiome from stool samples was analyzed before and after travel. Generally, a decrease in microbial diversity and an increase in Enterobacterales and, additionally, an acquisition of antibiotic-resistant strains were seen. During the study, approx. 67% of travellers acquired new *E. coli* strains that differed phylogenetically from their *E. coli* strains before travel. Interestingly, *E. coli* phylogroup A and B1 strains were more frequently acquired during travel; by contrast, phylogroup B2 and D strains were more commonly carried before travel. Furthermore, approx. 33% of travellers acquired an ESBL-producing Enterobacterales strain during travel, and fluoroquinolone resistance genes were detected in 97 travellers, with no carriage prior to travel. Additionally, a general increase in antibiotic resistance genes was also observed; a 56% higher abundance of resistance genes was detected in the intestinal microbiome after travel compared to that before [43].

It is important to mention that no specific microbiome signature or marker was detected that would have indicated an increased risk for the acquisition of any antibiotic-resistant strain during travel. This highlights that generally exogenous factors increase the risk of acquiring antibiotic-resistant bacteria during travel. These exogenous factors include travel to Asia and Africa, consumption of uncooked vegetables and other dietary factors and antibiotic use and diarrhea during travel [43].

## 6. Experimental Models in the Colonization of MDR *K. pneumoniae*

Several host factors in the intestinal tract can influence the successful colonization of MDR *K. pneumoniae* strains. The major endogenous factors are the level of IgA and alpha-defensin 5 and beta-defensin 3 production in the mucus layer. It has been demonstrated in a murine model that colonization with CTX-M-15- and OXA-162-producing *K. pneumoniae* ST15 clone differed from colonization with CTX-M-15-producing *E. coli* that carried IncF plasmids and from colonization with OXA-162-producing *E. coli* that carried an IncL plasmid compared to an *E. coli* strain that lacked all resistance determinants. There were differences in the production of IgA, alpha-defensin 5 and beta-defensin 3, namely, that in the case of colonization with CTX-M-15- and OXA-162-producing *K. pneumoniae*, CTX-M-15-producing *E. coli* and OXA-162-producing *E. coli*, in all groups, the IgA levels increased. However, in colonization with *E. coli*, no difference in IgA production was detected. The production of beta-defensin 3 increased in all groups; it reached the highest levels in colonization with CTX-M-15- and OXA-162-producing *K. pneumoniae* and CTX-M-15-producing *E. coli*. Alpha defensin 5 production increased in all groups. It is worth mentioning that the IgA level positively correlated with the colonizing bacterial cell count; however, alpha-defensin 5 production was inversely correlated with colonizing bacterial cell count and IgA levels. The presence of different plasmids also influenced colonization, as follows: the IncF plasmid enhanced the production of beta-defensin 3, and the bacterial count of the *Muribaculaceae* family correlated with the IncL plasmid [39].

On the microbiome level, the *Bacteroidota* phylum was dominant in groups where colonization was positive for CTX-M-15- and OXA-162-producing *K. pneumoniae*, CTX-M-15-producing *E. coli and* OXA-162-producing *E. coli*; however, in the case of colonization with *E. coli* lacking resistance genes, the *Proteobacteria* phylum was dominant. Interestingly, the *Muribaculaceae* family was more frequently detected during colonization with the antibiotic-resistant strains, including both *K. pneumoniae* and *E. coli*; however, in the case of colonization with *E. coli* lacking resistance determinants, the *Lachnospiraceae* family was dominant in the microbiota, indicating the protective role of *Lachnospiraceae* against the colonization of MDR *K. pneumoniae* and antibiotic-resistant *E. coli* [39].

Microbial communities in the intestinal tract interfere with colonization by MDR *K. pneumoniae*. The *Lachnospiraceae* family (e.g., *Lachnoclostridium*, *Roseburia*, *Anaerostipes*, *Tyzzerella*, *Agathobacter*) were negatively correlated with the colonization of MDR *K. pneumoniae* and reached a high abundance in the intestine where colonization with a non-MDR strain was present. By contrast, a high abundance of Enterobacterales indicated enhanced colonization with MDR *K. pneumoniae* [71].

During a study with a murine model, the impact of different antibiotics (ampicillin, ceftazidime, ciprofloxacin) was analyzed in correlation with intestinal colonization by MDR *K. pneumoniae*. The administration of ampicillin and ceftazidime increased the colonization of MDR *K. pneumoniae*; however, ciprofloxacin decreased the colonization bacterial cell count in the intestine. Furthermore, the gene copy number of *bla*_CTX-M-15_ beta-lactamase gene increased in the intestinally carried *K. pneumoniae* through the administration of ceftazidime [72].

## 7. Discussion

Intestinal colonization plays a central role in the dissemination of antibiotic-resistant strains, and among intestinally carried bacteria, MDR Enterobacterales strains are the most important, according to their medical importance. These MDR strains can asymptomatically colonize the gut, and later on these can serve as a source of infection. Generally, the rate of intestinally carried MDR Enterobacterales strains is increasing worldwide [12]. Several protocols have been set to decrease MDR colonization in the intestinal tract. Fecal microbiota transplantation (FMT) has been used in several countries to diminish the intestinal colonization of MDR Enterobacterales. However, the efficacy of FMT intervention is diverse, as can be explained by the fact that prior intestinal microbiome analysis is not always performed on the donor and on recipient fecal samples before FMT [73,74].

The intestinal tract is a diverse and dynamically changing environment that favours the colonization of different bacteria; furthermore, these bacteria can thrive in the intestine and take up different antibiotic resistance mechanisms that can be passed on [40]. However, based on the microbial community level, different correlations can be seen. The balance between the microbial communities in the gut can be influenced by exogenous and endogenous factors. The application of broad-spectrum antibiotics during hospitalization can disrupt the endogenous normal microbial environment in the intestine, leading to a decrease in microbial diversity; moreover, additional medical interventions can further enhance collateral damage, which leads to dysbiosis. The colonizer MDR strain itself and its resistance plasmids influence successful intestinal colonization. IncF and IncL plasmids that are usually present in MDR *K. pneumoniae* strains as well as small cryptic plasmids were detected in MDR *E. coli* and *K. pneumoniae* strains, and all can play a role in colonization and dynamic changes in the intestinal environment [39,40]. Through horizontal gene transfer, these plasmids can be exchanged between different bacterial species, thus enabling the transmission of antibiotic resistance genes (e.g., ESBLs) and other beneficial genetic markers for bacteria. Additionally, several other strain-related factors can further enhance colonization: the expression of type 3 fimbria through sensor histidine kinase CpxA in *K. pneumoniae* and the Type VI Secretion System (T6SS) can also trigger the colonization of *K. pneumoniae* in the intestine [41,42].

On the other hand, several protective factors have been analyzed that can diminish the damage in normal microbial communities, such as mucosal factors (e.g., IgA, defensin production) and host immune status. On the microbiome level, different microbial communities have a role in intestinal colonization with MDR Enterobacterales strains. A high abundance of *Lachnospiraceae*, *Dorea*, *Atopobiaceae*, *F. prausnitzii*, *Collinsella aerofaciens*, *Roseburia* and *Tyzzerella* was detected in association with no colonization with MDR Enterobacterales strains. In contrast, a high abundance of Enterobacterales enhanced the colonization of MDR Enterobacterales strains, which is reasonable when taking into account that taxonomically closely related bacterial species can exchange resistance genes with high frequency among themselves, and the higher abundance of Enterobacterales in the gut will enable a higher number of recipient bacterial cells, enhancing a longer term intestinal presence of the MDR strains.

Limitations of the current literature include a lack of standardization in microbiome sequencing and analysis, differences between observational and experimental studies and the need to distinguish correlation from causation.

## 8. Conclusions

Overall, the intestinal colonization of MDR Enterobacterales strains is a multifactorial process that correlates with different microbial communities and it is influenced by several endogenous and exogenous factors. In the intestinal tract, a high abundance of the Lachnospiraceae family indicates no colonization with MDR strains; by contrast, a high abundance of Enterobacterales indicates a higher carriage rate of ESBL Enterobacterales. Hospitalization, exposure to antibiotics and dysbiosis enhance intestinal colonization with MDR Enterobacterales strains.

## 9. Future Directions

Analysis of the intestinal microbiome offers several possibilities for exploring long- and short-term colonization with MDR bacterial strains. The interactions between intestinal microbial communities can explain further correlations with other MDR bacteria, not just Enterobacterales strains. Controlled trials of fecal microbiota transplantation or next-generation probiotics for decolonization are needed. The exploration of bacteriophage or CRISPR-based strategies targeting MDR plasmids would offer a modern method to decrease MDR colonization. Randomized clinical trials are needed to test decolonization strategies by FMT, probiotics, phages and CRISPR-Cas. Longitudinal multi-omics studies are also needed to disentangle cause–effect relationships between antibiotic exposure, microbiome shifts and MDR acquisition. The development of predictive models incorporating microbiome signatures and host factors is needed to guide infection-control policies.

## Figures and Tables

**Table 1:** Intestinal colonization with MDR Enterobacterales strains in different patient groups and in different countries. (ESBL: extended-spectrum beta-lactamase; HSCT: hematopoietic stem-cell-transplanted patients; ICU: intensive care unit.)

Strain	Antibiotic Resistance	No. of MDR Strains	Overall Colonization Rate	Country	Year	Reference
***K. pneumoniae***	ESBL, carbapenem resistant	17	17/386 (4.4%) hospitalized patients	USA	2017–2018	[28]
***K. pneumoniae***	KPC	37	37/243 (15.2%) ICU patients	China	2018	[29]
***E. coli***	ESBL	16/29	29/524 (5.5%) ICU patients	France	2014–2015	[17]
***K. pneumoniae***	ESBL	7/29				
***Enterobacter*** ** spp.**	ESBL	5/29				
***Citrobacter sedlakii***	ESBL	1/29				
***E. coli***	ESBL	10/27	27/93 (29%) patients at a Dutch nursing home	The Netherlands	2016–2017	[30]
***Enterobacter cloacae***	ESBL	3/27				
***Citrobacter non-koseri***	ESBL	1/27				
***E. coli***	fluoroquinolone, aminoglycoside-resistant	13/27				
***E. coli***	ESBL *n* = 198 (44 CTX-M type)	198	198/2751 (7%) Dutch adults	The Netherlands	2016–2017	[31]
***E. coli***	ESBL	22/24	24/302 (7.9%) ICU patients	Switzerland	2014–2015	[16]
***K. pneumoniae***	ESBL	1/24				
***Providentia stuartii***	ESBL	1/24				
***K. pneumoniae***	CTX-M-15	13/14	14/335 (4%) newborns	Sweden	2008–2009	[32]
***E. coli***	CTX-M-15	1/14				
***K. pneumoniae***	ESBL	4/59	59/187 (31%) adults at long term care-facility	Spain	2018–2019	[33]
***E. coli***	ESBL	55/59				
***K. pneumoniae***	OXA-48	31/76	76/254 (30%) surgical ICU	Spain	2012–2013	[18]
**Enterobacterales**	ESBL	45/76				
**Enterobacterales**	ESBL	11/48	11/48 (23%) HSCT patients	Italy	2017–2020	[34]
***K. pneumoniae***	KPC	45/88	88/350 (25%) newborns	Serbia	2018–2019	[35]
***K. pneumoniae***	OXA-48	42/88				
***E. coli***	NDM	1/88				
***K. pneumoniae***	KPC	4/81	81/500 (16%) hospitalized patients	Türkiye	2021–2023	[36]
***K. pneumoniae***	NDM	6/81				
***K. pneumoniae***	OXA-48	14/81				
***E. coli***	NDM	1/81				
***E. coli***	OXA-48	5/81				
***K. pneumoniae***	NDM	40/55	55/211 (26%) HSCT patients	Tunisia	2015–2019	[37]
***E. coli***	OXA-48	8/55				
***E. coli***	ESBL	89/134	134/215 (62%) ICU patients	Thailand	2014–2015	[15]
***K. pneumoniae***	ESBL	25/134				
***E. coli***	ESBL	10/27	27/137 (19.7%) ICU patients	Lao PDR	2019	[38]
***K. pneumoniae***	ESBL	12/27				
***K. pneumoniae***	carbapenem resistant	5/27				

## Data Availability

No new data were created or analyzed in this study. Data sharing is not applicable to this article.

## References

[1] Wuethrich I., W. Pelzer B., Khodamoradi Y., Vehreschild M.J.G.T. (2021). The role of the human gut microbiota in colonization and infection with multidrug-resistant bacteria. Gut Microbiome.

[2] Isles N.S., Mu A., Kwong J.C., Howden B.P., Stinear T.P. (2022). Gut microbiome signatures and host colonization with multidrug-resistant bacteria. Trends Microbiol..

[3] Ayobami O., Brinkwirth S., Eckmanns T., Markwart R. (2022). Antibiotic resistance in hospital-acquired ESKAPE-E infections in low- and lower-middle-income countries: A systematic review and meta-analysis. Emerg. Microbes Infect..

[4] Cassini A., Högberg L.D., Plachouras D., Quattrocchi A., Hoxha A., Simonsen G.S., Colomb-Cotinat M., Kretzschmar M.E., Devleesschauwer B., Cecchini M. (2019). Burden of AMR Collaborative Group. Attributable deaths and disability-adjusted life-years caused by infections with antibiotic-resistant bacteria in the EU and the European Economic Area in 2015: A population-level modelling analysis. Lancet Infect. Dis..

[5] GBD 2021 Antimicrobial Resistance Collaborators (2024). Global burden of bacterial antimicrobial resistance 1990–2021: A systematic analysis with forecasts to 2050. Lancet.

[6] (2024). WHO Bacterial Priority Pathogens List, 2024: Bacterial Pathogens of Public Health Importance to Guide Research, Development and Strategies to Prevent and Control Antimicrobial Resistance.

[7] Krul D., Negoseki B.R.D.S., Siqueira A.C., Tomaz A.P.O., Dos Santos É.M., de Sousa I., Vasconcelos T.M., Marinho I.C.R., Arend L.N.V.S., Mesa D. (2025). Spread of antimicrobial-resistant clones of the *ESKAPEE* group: From the clinical setting to hospital effluent. Sci. Total Environ..

[8] Romdhani A., Cheriet S., Abbassi M.S., Lengliz S., Hynds P., Boutiba-Ben Boubaker I., Landolsi R.B. (2023). High-risk clonal lineages among extended-spectrum beta-lactamase producing *Escherichia coli* and *Klebsiella pneumoniae* from urban and rural stagnant water samples in Tunisia. Acta Microbiol. Immunol. Hung..

[9] Le Guern R., Stabler S., Gosset P., Pichavant M., Grandjean T., Faure E., Karaca Y., Faure K., Kipnis E., Dessein R. (2021). Colonization resistance against multi-drug-resistant bacteria: A narrative review. J. Hosp. Infect..

[10] Woelfel S., Silva M.S., Stecher B. (2024). Intestinal colonization resistance in the context of environmental, host, and microbial determinants. Cell Host Microbe.

[11] Liao W., Huang N., Zhang Y., Sun Y., Chen T., Zheng W., Chen L., Wen H., Cao J., Zhou T. (2021). Comparison of Carbapenem-Resistant *Klebsiella pneumoniae* Strains Causing Intestinal Colonization and Extraintestinal Infections: Clinical, Virulence, and Molecular Epidemiological Characteristics. Front. Public Health.

[12] Bezabih Y.M., Bezabih A., Dion M., Batard E., Teka S., Obole A., Dessalegn N., Enyew A., Roujeinikova A., Alamneh E. (2022). Comparison of the global prevalence and trend of human intestinal carriage of ESBL-producing *Escherichia coli* between healthcare and community settings: A systematic review and meta-analysis. JAC Antimicrob. Resist..

[13] Bezabih Y.M., Sabiiti W., Alamneh E., Bezabih A., Peterson G.M., Bezabhe W.M., Roujeinikova A. (2021). The global prevalence and trend of human intestinal carriage of ESBL-producing *Escherichia coli* in the community. J. Antimicrob. Chemother..

[14] Campos-Madueno E.I., Moradi M., Eddoubaji Y., Shahi F., Moradi S., Bernasconi O.J., Moser A.I., Endimiani A. (2023). Intestinal colonization with multidrug-resistant *Enterobacterales*: Screening, epidemiology, clinical impact, and strategies to decolonize carriers. Eur. J. Clin. Microbiol. Infect. Dis..

[15] Kiddee A., Assawatheptawee K., Na-Udom A., Boonsawang P., Treebupachatsakul P., Walsh T.R., Niumsup P.R. (2019). Risk factors for extended-spectrum beta-lactamase-producing *Enterobacteriaceae* carriage in patients admitted to intensive care unit in a tertiary care hospital in Thailand. Microb. Drug Resist..

[16] Emmanuel Martinez A., Widmer A., Frei R., Pargger H., Tuchscherer D., Marsch S., Egli A., Tschudin-Sutter S. (2019). ESBL colonization at ICU admission: Impact on subsequent infection, carbapenem-consumption, and outcome. Infect. Control Hosp. Epidemiol..

[17] Jalalzai W., Boutrot M., Guinard J., Guigon A., Bret L., Poisson D.M., Boulain T., Barbier F. (2018). Cessation of screening for intestinal carriage of extended-spectrum beta-lactamase-producing *Enterobacteriaceae* in a low-endemicity intensive care unit with universal contact precautions. Clin. Microbiol. Infect..

[18] Maseda E., Salgado P., Anillo V., Ruiz-Carrascoso G., Gomez-Gil R., Martin-Funke C., Gimenez M.J., Granizo J.J., Aguilar L., Gilsanz F. (2017). Risk factors for colonization by carbapenemase producing enterobacteria at admission to a Surgical ICU: A retrospective study. Enferm. Infecc. Microbiol. Clin..

[19] Milic M., Siljic M., Cirkovic V., Jovicevic M., Perovic V., Markovic M., Martic J., Stanojevic M., Mijac V. (2021). Colonization with multidrug-resistant bacteria in the first week of life among hospitalized preterm neonates in Serbia risk factors and outcomes. Microorganisms.

[20] Arhoune B., El Fakir S., Himri S., Moutaouakkil K., El Hassouni S., Benboubker M., Hmami F., Oumokhtar B. (2021). Intense intestinal carriage and subsequent acquisition of multidrug-resistant enterobacteria in neonatal intensive care unit in Morocco. PLoS ONE.

[21] Xu Q., Pan F., Sun Y., Wang C., Shi Y., Zhang T., Yu F., Zhang H. (2020). Fecal carriage and molecular epidemiology of Carbapenem-resistant *Enterobacteriaceae* from inpatient children in a pediatric hospital of Shanghai. Infect. Drug Resist..

[22] Harbaoui S., Ferjani S., Abbassi M.S., Saidani M., Gargueh T., Ferjani M., Hammi Y., Boutiba-Ben Boubaker I. (2022). Genetic heterogeneity and predominance of *bla*_CTX-M-15_ in cefotaxime resistant *Enterobacteriaceae* isolates colonizing hospitalized children in Tunisia. Lett. Appl. Microbiol..

[23] Schaumburg F., Alabi A., Kokou C., Grobusch M.P., Kock R., Kaba H., Becker K., Adegnika A.A., Kremsner P.G., Peters G. (2013). High burden of extended-spectrum beta-lactamase-producing *Enterobacteriaceae* in Gabon. J. Antimicrob. Chemother..

[24] van Aartsen J.J., Moore C.E., Parry C.M., Turner P., Phot N., Mao S., Suy K., Davies T., Giess A., Sheppard A.E. (2019). Epidemiology of paediatric gastrointestinal colonisation by extended spectrum cephalosporin-resistant *Escherichia coli* and *Klebsiella pneumoniae* isolates in north-west Cambodia. BMC Microbiol..

[25] Kibwana U.O., Manyahi J., Sandnes H.H., Blomberg B., Mshana S.E., Langeland N., Moyo S.J. (2022). Gastrointestinal colonization of extended-spectrum beta-lactamase-producing bacteria among children below five years of age hospitalized with fever in Dar es Salaam, Tanzania. J. Glob. Antimicrob. Resist..

[26] Mahjoub Khachroub A., Souguir M., Châtre P., Elhouda Bouhlel N., Jaidane N., Drapeau A., El Kantaoui M., Azaiez S., Madec J.Y., Mansour W. (2024). Carriage Rate of *Enterobacterales* Resistant to Extended-Spectrum Cephalosporins in the Tunisian Population. Pathogens.

[27] Saidel-Odes L., Sagi O., Troib S., Leeman H., Nativ R., Schlaeffer-Yosef T., Azulay H., Nesher L., Borer A. (2024). Risk Factors and Outcomes of Patients Colonized with KPC and NDM Carbapenemase-Producing *Enterobacterales*. Antibiotics.

[28] Rao K., Patel A., Sun Y., Vornhagen J., Motyka J., Collingwood A., Teodorescu A., Baang J.H., Zhao L., Kaye K.S. (2021). Risk Factors for Klebsiella Infections among Hospitalized Patients with Preexisting Colonization. mSphere.

[29] Qin X., Wu S., Hao M., Zhu J., Ding B., Yang Y., Xu X., Wang M., Yang F., Hu F. (2020). The Colonization of Carbapenem-Resistant *Klebsiella pneumoniae*: Epidemiology, Resistance Mechanisms, and Risk Factors in Patients Admitted to Intensive Care Units in China. J. Infect. Dis..

[30] Ducarmon Q.R., Terveer E.M., Nooij S., Bloem M.N., Vendrik K.E.W., Caljouw M.A.A., Sanders I.M.J.G., van Dorp S.M., Wong M.C., Zwittink R.D. (2021). Microbiota-associated risk factors for asymptomatic gut colonisation with multi-drug-resistant organisms in a Dutch nursing home. Genome Med..

[31] Ducarmon Q.R., Zwittink R.D., Willems R.P.J., Verhoeven A., Nooij S., van der Klis F.R.M., Franz E., Kool J., Giera M., Vandenbroucke-Grauls C.M.J.E. (2022). Gut colonisation by extended-spectrum beta-lactamase-producing *Escherichia coli* and its association with the gut microbiome and metabolome in Dutch adults: A matched case-control study. Lancet Microbe.

[32] Nordberg V., Jonsson K., Giske C.G., Iversen A., Aspevall O., Jonsson B., Camporeale A., Norman M., Navér L. (2018). Neonatal intestinal colonization with extended-spectrum beta-lactamase-producing *Enterobacteriaceae*-a 5-year follow-up study. Clin. Microbiol. Infect..

[33] Colmenarejo C., Rodríguez-Jiménez C., Navarro F.J., Mateo A.B., Pellejero E.M., Belda-Moreno R.M., Ureña-Méndez R., Pérez-Serrano R., Illescas S., Muñoz-Rodríguez J.R. (2025). One-year monitorization of the gut colonization by multidrug resistant bacteria in elderly of a single long-term care facility. JAC Antimicrob. Resist..

[34] Corcione S., Ferrocino I., Lupia T., Busca A., Bianco G., Dellacasa C., Giaccone L., Brunello L., Butera S., Costa C. (2025). Influence of ESBL colonization status on gut microbiota composition during allogenic hematopoietic stem cell transplantation. Sci. Rep..

[35] Mijac V., Brkic S., Milic M., Siljic M., Cirkovic V., Perovic V., Markovic M., Cirkovic I., Stanojevic M. (2023). Intestinal Colonization of Preterm Neonates with Carbapenem Resistant Enterobacteria at Hospital Discharge. Antibiotics.

[36] Sanli K., Öncel B. (2025). Analysis of rectal carbapenem-resistant Enterobactericeae colonization results first report in Istanbul/Turkiye: *Klebsiella pneumoniae* co-producing *bla*_KPC_ + *bla*_NDM_ + *bla*_OXA-48_ in a single strain. BMC Infect. Dis..

[37] Ayari I., Chebbi Y., Raddaoui A., Belloumi D., Frigui S., Werhni R., Ben Othmen T., Abedejlil N., Achour W. (2024). High rates of intestinal colonization with carbapenemase producing *Enterobacteriaceae* in hematopoietic stem cell transplant recipients. Acta Microbiol. Immunol. Hung..

[38] Sewunet T., Sriram K.K., Nguyen H.H., Sithivong N., Hoang N.T.B., Sychareun V., Nengmongvang K., Larsson M., Olson L., Westerlund F. (2022). Fecal carriage and clonal dissemination of *bla*_NDM-1_ carrying *Klebsiella pneumoniae* sequence type 147 at an intensive care unit in Lao PDR. PLoS ONE.

[39] Stercz B., Domokos J., Dunai Z.A., Makra N., Juhasz J., Ostorhazi E., Kocsis B., Szabo D. (2024). The Roles of a Multidrug-Resistant *Klebsiella pneumoniae* High-Risk Clone and Its Resistance Plasmids on the Gastrointestinal Colonization and Host-Defense Effectors in the Gut. Antibiotics.

[40] Brolund A., Rajer F., Giske C.G., Melefors Ö., Titelman E., Sandegren L. (2019). Dynamics of Resistance Plasmids in Extended-Spectrum-beta-Lactamase-Producing *Enterobacteriaceae* during Postinfection Colonization. Antimicrob. Agents Chemother..

[41] Li D., Shi Q., He L., Luo J., Zhu H., Hua X., Yu Y., Jiang Y., Tao L. (2025). Cpx-mediated amino acid sensing diversifies gastrointestinal colonization of *Klebsiella pneumoniae*. mLife.

[42] Zhao C., Liu P., Lin X., Wan C., Liao K., Guo P., Deng J., Wu Z., Peng Y., Huang J. (2025). The type VI secretion system as a potential predictor of subsequent bloodstream infection of carbapenem-resistant *Klebsiella pneumoniae* strains on intestinal colonization. Infection.

[43] Worby C.J., Sridhar S., Turbett S.E., Becker M.V., Kogut L., Sanchez V., Bronson R.A., Rao S.R., Oliver E., Walker A.T. (2023). Gut microbiome perturbation, antibiotic resistance, and *Escherichia coli* strain dynamics associated with international travel: A metagenomic analysis. Lancet Microbe.

[44] Peña-Durán E., García-Galindo J.J., López-Murillo L.D., Huerta-Huerta A., Balleza-Alejandri L.R., Beltrán-Ramírez A., Anaya-Ambriz E.J., Suárez-Rico D.O. (2025). Microbiota and Inflammatory Markers: A Review of Their Interplay, Clinical Implications, and Metabolic Disorders. Int. J. Mol. Sci..

[45] Mann E.R., Lam Y.K., Uhlig H.H. (2024). Short-chain fatty acids: Linking diet, the microbiome and immunity. Nat. Rev. Immunol..

[46] Arbas S.M., Narayanasamy S., Herold M., Lebrun L.A., Hoopmann M.R., Li S. (2021). Roles of bacteriophages, plasmids and CRISPR immunity in microbial community dynamics revealed using time-series integrated meta-omics. Nat. Microbiol..

[47] Wende M., Osbelt L., Eisenhard L., Lesker T.R., Damaris B.F., Mutukumarasamy U., Bielecka A., Almási É.D.H., Winter K.A., Schauer J. (2025). Suppression of gut colonization by multidrug-resistant *Escherichia coli* clinical isolates through cooperative niche exclusion. Nat. Commun..

[48] Mahamat O.O., Tidjani A., Lounnas M., Hide M., Benavides J., Somasse C., Ouedraogo A.-S., Sanou S., Carrière C., Bañuls A.-L. (2019). Fecal carriage of extended-spectrum β-lactamase-producing *Enterobacteriaceae* in hospital and community settings in Chad. Antimicrob. Resist. Infect. Control.

[49] Ko Y.J., Moon H.-W., Hur M., Park C.-M., Cho S.E., Yun Y.-M. (2013). Fecal carriage of extended-spectrum β-lactamase-producing *Enterobacteriaceae* in Korean community and hospital settings. Infection.

[50] Kurz M.S.E., Bayingana C., Ndoli J.M., Sendegeya A., Durst A., Pfüller R., Gahutu J.B., Mockenhaupt F.P. (2017). Intense pre-admission carriage and further acquisition of ESBL-producing *Enterobacteriaceae* among patients and their caregivers in a tertiary hospital in Rwanda. Trop. Med. Int. Health.

[51] van den Bunt G., van Pelt W., Hidalgo L., Scharringa J., de Greeff S.C., Schürch A.C., Mughini-Gras L., Bonten M.J.M., Fluit A.C. (2019). Prevalence, risk factors and genetic characterisation of extended-spectrum β-lactamase and carbapenemase-producing *Enterobacteriaceae* (ESBL-E and CPE): A community-based cross-sectional study, The Netherlands, 2014 to 2016. Euro Surveill..

[52] Arnan M., Gudiol C., Calatayud L., Liñares J., Dominguez M.Á., Batlle M., Ribera J.M., Carratalà J., Gudiol F. (2011). Risk factors for, and clinical relevance of, faecal extended-spectrum β-lactamase producing *Escherichia coli* (ESBL-EC) carriage in neutropenic patients with haematological malignancies. Eur. J. Clin. Microbiol. Infect. Dis..

[53] van Loon K., Voor in ‘t holt A.F., Vos M.C. (2017). A systematic review and meta-analyses of the clinical epidemiology of carbapenem-resistant *Enterobacteriaceae*. Antimicrob. Agents Chemother..

[54] Gorrie C.L., Mirčeta M., Wick R.R., Edwards D.J., Thomson N.R., Strugnell R.A., Pratt N.F., Garlick J.S., Watson K.M., Pilcher D.V. (2017). Gastrointestinal carriage is a major reservoir of *Klebsiella pneumoniae* infection in intensive care patients. Clin. Infect. Dis..

[55] Martin R.M., Bachman M.A. (2018). Colonization, infection, and the accessory genome of *Klebsiella pneumoniae*. Front. Cell Infect. Microbiol..

[56] Martin R.M., Cao J., Brisse S., Passet V., Wu W., Zhao L., Malani P.N., Rao K., Bachman M.A., Castanheira M. (2016). Molecular epidemiology of colonizing and infecting isolates of *Klebsiella pneumoniae*. mSphere.

[57] Loukili N.H., Perrin A., Gaillot O., Bruandet A., Boudis F., Sendid B., Nseir S., Zahar J.-R. (2025). Is intestinal colonization with multidrug-resistant *Enterobacterales* associated with higher rates of nosocomial *Enterobacterales* bloodstream infections?. Int. J. Infect. Dis..

[58] Rettedal S., Lohr I.H., Bernhoff E., Natas O.B., Sundsfjord A., Oymar K. (2015). Extended-spectrum beta-lactamase producing *Enterobacteriaceae* among pregnant women in Norway: Prevalence and maternal-neonatal transmission. J. Perinatol..

[59] Cassettari V.C., da Silveira I.R., Dropa M., Lincopan N., Mamizuka E.M., Matte M.H., Matté G., Menezes P. (2009). Risk factors for colonisation of newborn infants during an outbreak of extended-spectrum beta-lactamase producing *Klebsiella pneumoniae* in an intermediate-risk neonatal unit. J. Hosp. Infect..

[60] Titelman E., Hasan C.M., Iversen A., Naucler P., Kais M., Kalin M., Giske C.G. (2014). Faecal carriage of extended-spectrum ß-lactamase-producing *Enterobacteriaceae* is common 12 months after infection and is related to strain factors. Clin. Microbiol. Infect..

[61] Detsis M., Karanika S., Mylonakis E. (2017). ICU Acquisition Rate, Risk Factors, and Clinical Significance of Digestive Tract Colonization with Extended-Spectrum Beta-Lactamase-Producing *Enterobacteriaceae*: A Systematic Review and Meta-Analysis. Crit. Care Med..

[62] Carvalho A.S., Lagana D., Catford J., Shaw D., Bak N. (2020). Bloodstream infections in neutropenic patients with haematological malignancies. Infect. Dis. Health.

[63] McMahon S., Sahasrabhojane P., Kim J., Franklin S., Chang C.C., Jenq R.R., Hillhouse A.E., Shelburne S.A., Galloway-Peña J. (2023). Contribution of the Oral and Gastrointestinal Microbiomes to Bloodstream Infections in Leukemia Patients. Microbiol. Spectr..

[64] Stoma I., Littmann E.R., Peled J.U., Giralt S., van den Brink M.R.M., Pamer E.G., Taur Y. (2021). Compositional flux within the intestinal microbiota and risk for bloodstream infection with Gram-negative bacteria. Clin. Infect. Dis..

[65] Annavajhala M.K., Gomez-Simmonds A., Macesic N., Sullivan S.B., Kress A., Khan S.D., Giddins M.J., Stump S., Kim G.I., Narain R. (2019). Colonizing multidrug-resistant bacteria and the longitudinal evolution of the intestinal microbiome after liver transplantation. Nat. Commun..

[66] Ny S., Lofmark S., Borjesson S., Englund S., Ringman M., Bergstrom J., Naucler P., Giske C.G., Byfors S. (2017). Community carriage of ESBL-producing *Escherichia coli* is associated with strains of low pathogenicity: A Swedish nationwide study. J. Antimicrob. Chemother..

[67] Swedres-Svarm (2017). Consumption of Antibiotics and Occurrence of Resistance in Sweden.

[68] Arcilla M.S., van Hattem J.M., Haverkate M.R., Bootsma M.C.J., van Genderen P.J.J., Goorhuis A., Grobusch M.P., Lashof A.M.O., Molhoek N., Schultsz C. (2017). Import and spread of extended-spectrum beta-lactamase-producing *Enterobacteriaceae* by international travellers (COMBAT study): A prospective, multicentre cohort study. Lancet Infect. Dis..

[69] Kuenzli E., Jaeger V.K., Frei R., Neumayr A., DeCrom S., Haller S., Blum J., Widmer A.F., Furrer H., Battegay M. (2014). High colonization rates of extended-spectrum b-lactamase (ESBL)-producing *Escherichia coli* in Swiss travellers to South Asia—A prospective observational multicentre cohort study looking at epidemiology, microbiology and risk factors. BMC Infect. Dis..

[70] Dall L.B., Lausch K.R., Gedebjerg A., Fuursted K., Storgaard M., Larsen C.S. (2019). Do probiotics prevent colonization with multi-resistant *Enterobacteriaceae* during travel? A randomized controlled trial. Travel Med. Infect. Dis..

[71] Juhász J., Ligeti B., Gajdács M., Makra N., Ostorházi E., Farkas F.B., Stercz B., Tóth Á., Domokos J., Pongor S. (2021). Colonization Dynamics of Multidrug-Resistant *Klebsiella pneumoniae* Are Dictated by Microbiota-Cluster Group Behavior over Individual Antibiotic Susceptibility: A Metataxonomic Analysis. Antibiotics.

[72] Stercz B., Farkas F.B., Tóth Á., Gajdács M., Domokos J., Horváth V., Ostorházi E., Makra N., Kocsis B., Juhász J. (2021). The influence of antibiotics on transitory resistome during gut colonization with CTX-M-15 and OXA-162 producing *Klebsiella pneumoniae* ST15. Sci. Rep..

[73] Tacconelli E., Mazzaferri F., de Smet A.M., Bragantini D., Eggimann P., Huttner B.D., Kuijper E.J., Lucet J.C., Mutters N.T., Sanguinetti M. (2019). ESCMID-EUCIC clinical guidelines on decolonization of multidrug-resistant Gram-negative bacteria carriers. Clin. Microbiol. Infect..

[74] Zhang H.J., Wang H.W., Tian F.Y., Yang C.Z., Zhao M., Ding Y.X., Wang X.Y., Cui X.Y. (2024). Decolonization strategies for ESBL-producing or carbapenem-resistant *Enterobacterales* carriage: A systematic review and meta-analysis. Sci. Rep..

